# Right Heart Changes Impact on Clinical Phenotype of Amyloid Cardiac Involvement: A Single Centre Study

**DOI:** 10.3390/life10100247

**Published:** 2020-10-18

**Authors:** Sebastiano Cicco, Antonio Giovanni Solimando, Roberta Buono, Nicola Susca, Gianfranco Inglese, Assunta Melaccio, Marcella Prete, Roberto Ria, Vito Racanelli, Angelo Vacca

**Affiliations:** 1 Unit of Internal Medicine “Guido Baccelli”, Department of Biomedical Sciences and Human Oncology, University of Bari Aldo Moro Medical School, Piazza Giulio Cesare 11, I-70124 Bari, Italy; sebacicco@gmail.com (S.C.); antoniogiovannisolimando@gmail.com (A.G.S.); r.buono87@gmail.com (R.B.); susnic2@gmail.com (N.S.); gian.inglese1@gmail.com (G.I.); assuntamel@hotmail.it (A.M.); marcella.prete@uniba.it (M.P.); roberto.ria@uniba.it (R.R.); vito.racanelli1@uniba.it (V.R.); 2 Internal Medicine Department, AUO Policlinico Ospedali Riuniti, Viale L. Pinto, I-71122 Foggia, Italy; 3 IRCCS Istituto Tumori “Giovanni Paolo II” of Bari, Viale Orazio Flacco 65, I-70124 Bari, Italy

**Keywords:** amyloidosis, right heart, cardiac involvement, heart ultrasound

## Abstract

Amyloidosis is due to deposition of an excessive amount of protein in many parenchymal tissues, including myocardium. The onset of cardiac Amyloidosis (CA) is an inauspicious prognostic factor, which can lead to sudden death. We retrospectively analyzed 135 patients with systemic amyloidosis, admitted to our ward between 1981 and 2019. Among them, 54 patients (46.30% F/53.70% M, aged 63.95 ± 12.82) presented CA at baseline. In 53 patients, it was associated with a multiorgan involvement, while in one there was a primary myocardial deposition. As a control group, we enrolled 81 patients (49.30% F/50.70% M, aged 58.33 ± 15.65) who did not meet the criteria for CA. In 44/54 of patients CA was associated with AL, 5/54 with AA and 3/54 of patients with ATTR, and in 1/54 AL was related to hemodialysis and in 1/54 to Gel-Amyloidosis. The most common AL type was IgG (28/44); less frequent forms were either IgA (7/44) or IgD (2/44), while seven patients had a λ free light chain form. The 32 AL with complete Ig were 31 λ-chain and just one k-chain. CA patients presented normal BP (SBP 118.0 ± 8.4 mmHg; DBP 73.8 ± 4.9 mmHg), while those with nCA had an increased proteinuria (*p* = 0.02). TnI and NT-proBNP were significantly increased compared to nCA (*p* = 0.031 and *p* = 0.047, respectively). In CA patients we found an increased LDH compared to nCA (*p* = 0.0011). CA patients were also found to have an increased interventricular septum thickness compared to nCA (*p* = 0.002), a decreased Ejection Fraction % (*p* = 0.0018) and Doppler velocity E/e’ ratio (*p* = 0.0095). Moreover, CA patients had an enhanced right atrium area (*p* = 0.0179), right ventricle basal diameter (*p* = 0.0112) and wall thickness (*p* = 0.0471) compared to nCA, and an increased inferior cava vein diameter (*p* = 0.0495) as well. TAPSE was the method chosen to evaluate systolic function of the right heart. In CA subjects very poor TAPSE levels were found compared to nCA patients (*p* = 0.0495). Additionally, we found a significant positive correlation between TAPSE and lymphocyte count (r = 0.47; *p* = 0.031) as well as Gamma globulins (r = 0.43, *p* = 0.033), Monoclonal components (r = 0.72; *p* = 0.047) and IgG values (r = 0.62, *p* = 0.018). Conversely, a significant negative correlation with LDH (r = −0.57, *p* = 0.005), IVS (r = −0.51, *p* = 0.008) and diastolic function evaluated as E/e’ (r = −0.60, *p* = 0.003) were verified. CA patients had very poor survival rates compared to controls (30 vs. 66 months in CA vs. nCA, respectively, *p* = 0.15). Mean survival of CA individuals was worse also when stratified according to NT-proBNP levels, using 2500 pg/mL as class boundary (174 vs. 5.5 months, for patients with lower vs. higher values than the median, respectively *p* = 0.013). In much the same way, a decreased right heart systolic function was correlated with a worse prognosis (18.0 months median survival, not reached in subjects with lower values than 18 mm, *p* = 0.0186). Finally, our data highlight the potential prognostic and predictive value of right heart alterations characterizing amyloidosis, as a novel clinical parameter correlated to increased LDH and immunoglobulins levels. Overall, we confirm the clinical relevance of cardiac involvement suggests that right heart evaluation may be considered as a new marker for clinical risk stratification in patients with amyloidosis.

## 1. Introduction

The term “Amyloidosis” includes a group of protein folding disorders in which there is a deposition of an insoluble protein material that Rudolph Virchow in 1854 called “amyloid substance”. Stacked protein monomers are rich in β-sheets. They form proto-filaments measuring 2 to 5 nm in diameter. These filaments bind each other through hydrogen bonds creating complex insoluble polymers [1] resulting in Amyloid deposits. The classification identifies amyloidosis according to the nature of the main amyloid precursor protein. Up to 28 different proteins have currently been recognized to be amyloidogenic in humans [2,3].

The most common form of systemic amyloidosis in western countries is derived from the light chains of Immunoglobulin (AL amyloidosis) (about 85% of all newly diagnosed cases of amyloidosis), with an estimated incidence of 0.8 per 100,000 person/year and a prevalence of 40.5 cases per million in 2015 [3,4]. Rarely, it could be a consequence of heavy-chain immunoglobulins (AH). Like other “monoclonal gammopathies”, AL amyloidosis is a plasma cell dyscrasia, residing in a proliferating plasma cell clone that preserves the capacity of producing immunoglobulins and/or part of them. It has also been pointed out that 10–15% of myeloma patients develop AL amyloidosis.

The second most important type of acquired amyloidosis is the AA form, a rare complication of persistent inflammatory states. AA amyloid deposits are a consequence of an increase in N-terminal proteolytic fragments serum amyloid A (SAA) protein, which is an apolipoprotein of high-density lipoproteins (HDL) acting as an acute-phase reactant. During persistent chronic inflammatory disorders, sustained deposition of SAA occurs. In western countries, rheumatoid arthritis, inflammatory bowel disease and untreated familial Mediterranean fever are some of the diseases inducing the production of this secondary misfolding protein. On the contrary, in developing countries, infectious diseases such as tuberculosis, bronchiectasis and osteomyelitis are more commonly implicated [5]. Nowadays, hereditary amyloidosis is gaining ground. The above forms are a heterogeneous group of autosomal dominant, late-onset diseases caused by mutations in the genes coding for a set of plasma proteins, including transthyretin (ATTR), apolipoprotein A-I (apoA-I), apolipoprotein A-II, fibrinogen, gelsolin, cystatin C and lysozyme, which enrich the spectrum of amyloidogenic diseases. All the above inherited mutations lead to misfolded proteins, resulting in an enhanced tendency to proteolysis and remodeling, with an increased propensity to aggregation due to their β-sheet density, hydrophobicity and lack of electrochemical charge. Conversely, ATTR is one of the leading causes of cardiac Amyloidosis (CA), especially in elderly people [6].

From a clinical standpoint, cardiac involvement is very common in AL amyloidosis, involving up to 90% of patients during disease evolution [7,8], characterized by diastolic heart failure at the time of diagnosis in about 50% of cases. ATTR amyloid gradually influences the cardiac function over time; therefore, the clinical phenotype can often be asymptomatic until the amyloid involvement of the myocardium reaches the late disease stage [9].

Symptoms and signs are mostly non-specific, which is a further obstacle to making an early [10,11] diagnosis, complicated by the high prevalence of other cardiovascular diseases such as coronary heart disease, arrhythmias, and valvular heart diseases. In cases of a high clinical suspicion of a restrictive etiology of unexplained cardiomyopathy is essential. To obtain an accurate diagnosis and treatment a multidisciplinary approach is required.

Of note, the most commonly referred to symptoms of cardiac amyloidosis can be clinically associated with right heart (RH) failure, including atypical chest pain, dyspnea on exertion, fatigue, peripheral edema and palpitations.

**Figure 1:**
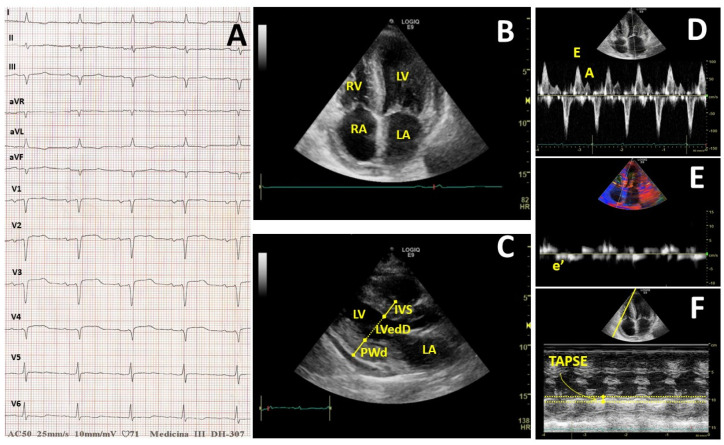
Heart involvement due to amyloid deposition will lead to different instrumental issues. One finding is low voltage diffused in all leads. (**A**) Other typical findings are echocardiography alterations. Increased thickness of the heart wall in bi-mode heart ultrasound both in the apical 4-chamber view (A4C) (**B**) and parasternal long-axis view (PLAx); (**C**) the latter is useful to evaluate left ventricle diameter (IVS, LVedD and PWd). A4C is useful to evaluate volumes; Doppler analysis was also performed in A4C: Pulsed wave Doppler on mitral valve (**D**) it was necessary to evaluate both E and A velocity, while tissue Doppler imaging (**E**) was used to measure e’ velocity; TAPSE was measured in A4C using monodimensional mode (**F**).

Electrocardiography shows the characteristic feature of low QRS voltage (Figure 1) in up to 50% of patients and, when coupled with the finding of increased ventricular wall thickness, it should prompt the suspicion of cardiac amyloidosis, given that left ventricular hypertrophy due to other causes shows high QRS voltages [12].

Left ventricular (LV) performance deteriorates in step with the disease progression. Characteristic echocardiography findings of cardiac amyloid represent a continuum of diastolic dysfunction repertories that progress from an impaired relaxation through a pseudonormal pattern to a restrictive pattern, due to the increased deposition of amyloid in the myocardium. Two-dimensional trans-thoracic echocardiography (2D-TTE) is a valuable tool for structural anatomy and ventricular function assessment in the diagnosis of cardiac amyloid. Echocardiographic studies have reported abnormalities in left ventricular (LV) function ranging from mild diastolic dysfunction with minimal other echocardiographic findings in early disease to LV hypertrophy and LV systolic dysfunction in late disease (Figure 1). However, the detection of disease at the above stages already indicates a significant cardiac amyloid involvement [13].

Echocardiographic evidence for a mean left ventricular wall thickness of >12 mm, in absence of other causes, and a tissue biopsy demonstrating amyloid at an alternative site might also be sufficient.

Current management of Cardiac Amyloidosis (CA) other than AL remains symptomatic, based on diuretics, cardiovascular medications and anticoagulation drugs when atrial fibrillation occurs. However, CA is the most frequent heart amyloid disease and negatively impacts patients’ prognosis, being characterized by a median survival of 6 months when untreated [4,14].

The aim of this case-control retrospective study was to evaluate the difference between patients who presented CA with the ones who did not (nCA). Moreover, we investigated the potential role played by both CA and the CA-related RH involvement in patients’ prognosis.

## 2. Methods

We retrospectively evaluated 166 consecutive patients discharged with a diagnosis of amyloidosis from our Internal Medicine unit between 1 January 1981 and 31 December 2019. We excluded patients who had no histological diagnosis, those without complete clinical records, and the ones who had neither undergone heart ultrasound nor the other necessary basic tests for diagnosis. We selected 135 patients who had detailed records of their clinical history available, at presentation and throughout follow-up.

Study patients underwent a complete assessment including physical examination at the first visit. Complete blood counts, erythrocyte sedimentation rate, C-reactive protein, liver and renal function tests and serum protein electrophoresis were carried out as baseline investigations in all patients. Additional tests included typing for immunocharacterization of monoclonal components, proteinuria and Bence–Jones. Free light chain (FLC) λ and κ monitoring on serum and urine was performed in 30 patients. All patients underwent electrocardiogram and echocardiography. Other instrumental exams, such as chest x-ray, chest computed tomography, ultrasound examination of the upper and lower abdomen, radiographs of the skeleton and magnetic resonance imaging of brain and spine were performed whenever an association between Amyloidosis and systemic diseases (such as multiple myeloma or lymphoma) was suspected or if the exam was deemed clinically useful. Echocardiography results were obtained on exams recorded over the years, performing a post-processing evaluation using parameters and methods already described elsewhere [15].

All patients provided written informed consent to take part in the study. The study protocol was approved by the Ethics Committee of the University of Bari Medical School (n.1587, dated 11 October 2017, by the Policlinico di Bari University Hospital) and conformed to the good clinical practice guidelines of the Italian Ministry of Health and the ethical guidelines of the Declaration of Helsinki, as revised and amended in 2004.

Amyloidosis was diagnosed by performing biopsy on periumbelical fat (109), rectum mucosa (7), heart (2), liver (7) and kidney (10). All patients underwent ultrasound evaluation of the heart, an electrocardiogram and laboratory tests to characterize the disease.

We questioned the Apulian regional patients’ database of the health care system Edotto© (Exprevia, Molfetta, Italy) to identify the date of death of patients who were lost to follow-up.

Data were plotted for the type of amyloid protein, organ involvement, and associated diseases. Then heart deposition was analyzed, as detailed in the consensus symposium [16,17]. In particular, we considered as affected by cardiac amyloidosis (CA) all the patients with an interventricular septum thickness greater than 12 mm, associated to at least one of the following patterns: low-voltage Electrocardiogram, Arrhythmias or pacemakers, diastolic dysfunction, heart failure.

Among all the patients, we selected the 54 who met the above-mentioned criteria for a diagnosis of CA (29 males and 25 females, aged 63.95 ± 12.82). Patients who did not meet the criteria used to define cardiac involvement in amyloidosis were classified as non-cardiac amyloidosis (nCA) and included in the control group (41 males and 40 females, aged 58.33 ± 15.65).

To analyze the distribution of dichotomous values we used the chi-squared test. Non-normally distributed data were analyzed using non-parametric statistics. The Mann–Whitney test was used for comparisons between the groups and the Spearman rank test for correlations. Normally distributed data were analyzed using parametric statistics; group comparisons were carried out using unpaired t tests. Correlation of normally distributed values was performed with the Pearson method. Survival comparison was made with the log-rank method (presented as Kaplan–Meier curves). *p*-values are shown for statistically significant differences, defined as *p* values < 0.05. Statistical analyses were performed using GraphPad Prism software, Version 8.0 (GraphPad Software, San Diego, CA, USA).

## 3. Results

**Figure 2:**
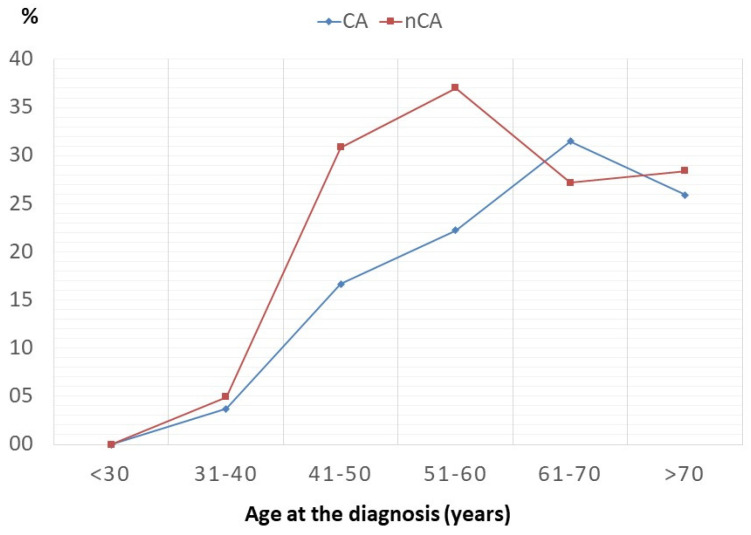
Age distribution.

In CA, amyloid deposition was associated with a systemic form of amyloidosis with multiorgan involvement in 53 patients, while just one had an amyloid localization only in the heart. Comparing incidence per age plotted per decade, the heart appears to be an involvement typically described in the elderly (Figure 2), although there is no difference in mean age between CA and nCA patients (Table 1). Groups were not significantly different also for Sex distribution (Table 1).

Most of the cases (44/54; 81.48%) had AL amyloidosis, while the 10 other cases included 5 AA amyloidosis, 3 ATTR, one Gel-amyloidosis and one dialysis-related Amyloidosis. CA had an increased incidence of AL compared to nCA (CA 81.48% vs. nCA 76.54%, *p* = ns) (Table 1). In C-AL patients, the amyloid clone produced mostly IgG (28/44 patients, 63.63%), IgA in 7 patients (15.90%), 2 patients had an IgD-related form (4.54%) and 7 presented a free λ chain-related amyloidosis (15.91%). In the 37 patients who presented an amyloidogenic complete immunoglobulin (Ig), 34 showed a λ chain and just three had a K chain associated Ig.

We observed that in 8/54 patients no disease was associated to heart involvement, while 13/54 presented a hematologic disease (Multiple Myeloma or MGUS), 4/54 a solid tumor (1 sarcoma, 1 thyroid neoplasia, 1 lung cancer and 1 HCC), 4/54 a connective tissue disorder (2 Sjogren’s Syndrome, 1 LES and 1 Rheumatoid Arthritis), 8/54 were affected by HCV and 25/54 presented other diseases (such as Diabetes, thyroid nodules, BPCO, arthrosis).

Bone marrow plasmacytosis was 12.2 ± 8.1% in all AL-CA: in Amyloidosis secondary to Myeloma plasmacytosis it was 22.5 ± 12.3% while in the remaining patients it was 2.1 ± 1.2%.

Other than heart, the organs most frequently involved were kidneys (32/54; 59.26%) and liver (20/54; 37.04%). Less frequent deposition was demonstrated in nodes or spleen (9/54 16.67%), the Peripheral Nervous System (PNS) (9/54; 16.67), skin (as purpura or erythema nodosum: 8/54; 14.81%), eyes (4/54; 7.41%), lung (5/54; 9.26%) and breast (1/54; 1.85%).

CA patients had normal blood pressure (BP): mean systolic BP was 118.0 ± 13.05 mmHg and diastolic BP was 72.92 ± 8.31 mmHg. Comparing values of CA patients to the nCA population, no significant differences were found for Hemoglobin (Hb), Mean corpuscular volume (MCV), white blood cells (WBC) while CA had a significantly decreased platelet (PLT) count (Table 2). There was no difference in Erythrocyte Sedimentation Rate (ESR), C-reactive protein (CRP) or Beta 2 microglobulin (B2M) (Table 2).

Immunological evaluation became necessary in many settings. The evaluation of lymphocyte to neutrophils (Neutrophil-to-Lymphocyte ratio: NLR) or platelets (Platelet-to-lymphocyte ratio: PLR) has been proposed as an indicator of the systemic inflammatory response that is widely investigated in cardiovascular diseases [18,19]. NLR was significantly increased in CA (CA 4.25 ± 3.63 vs. nCA 3.12 ± 2.05; *p* = 0.0307) while no difference was found in PLR (CA 183.1 ± 118.0 vs. nCA 164.4 ± 95.75; *p* = ns) (Table 2).

In AL amyloidosis, hematological tests were performed. CD20 positive lymphocyte percentages were increased in CA patients (Table 2). The monoclonal component (CM) was not significantly different as either percentage or absolute values (Table 2). Similarly, no significant difference was found in free light chain (FLC) in λ or k chain (Table 2).

Kidney impairment was not different in CA compared to nCA. In fact, no difference was found for creatinine and the estimated glomerular filtration rate evaluated with the EPI formula [20] (Table 3). Conversely, an increase in Blood Urea Nitrogen (BUN) was found in CA patients. Proteinuria was significantly increased in nCA (Table 3). There was no difference in blood electrolytes. Lactate dehydrogenase (LDH) was found to be significantly increased in CA. Furthermore, Troponin I vaues (TnI, CA 9.13 ± 5.79 vs. nCA 0.19 ± 0.37 ng/mL; *p* = 0.047) and the N-terminal fragment of brain natriuretic peptide (NT-proBNP, CA 8633.2 ± 2636.1 vs. nCA 1875.5 ± 850.8 pg/mL; *p* = 0.031) were significantly increased in CA compared to nCA patients (Table 3).

The electrocardiography (ECG) abnormalities found were Atrial Fibrillation (12/54), Extrasystoles (7/54), Q waves in two or more leads (5/54) and diffused low voltages (16/54), while 14 were considered to show a normal sinus rhythm.

Echocardiography results were interpreted considering both heart ultrasound guidelines [21] and heart involvement indicative of heart amyloidosis [22]. In CA, data indicate myocardial hypertrophy both of the Interventricular septum (IVS: 15.71 ± 3.21 mm) and posterior wall thickness (PWd; 15.39 ± 2.97 mm). Left ventricle interior end-diastolic diameter (LVedD) was reduced (43.80 ± 8.39 mm). Comparing the above data to the same measurement in nCA patients we found a significant difference in IVS (11.03 ± 2.04 mm, *p* < 0.0001), PWd (10.97 ± 2.06 mm, *p* < 0.0001), and LVedD (50.17 ± 4.32 mm, *p* = 0.0143). Consequently, there was a significant increase in relative wall thickness (RWT; CA 0.72 ± 0.29 vs. nCA 0.40 ± 0.15; *p* = 0.0022).

Left ventricle mass (LVM) was dramatically increased in CA in terms of the absolute value (CA 296.2 ± 112.5 vs. nCA 166.0 ± 108.1 gr; *p* = 0.015). Similarly, there was a significant increase in LVM when it was indexed for body surface area (LVMi; CA 184.4 ± 64.01 vs. nCA 129.9 ± 47.47 gr/m^2^; *p* = 0.013) and height elevated to 2.7 (LVMih^2,7^; CA 79.81 ± 27.10 vs. nCA 56.75 ± 25.06 gr/m^2,7^; *p* = 0.019).

Ejection fraction (EF%) was reduced in CA (CA 52.50 ± 10.58 vs. ns CA 60.94 ± 4.44%, *p* = 0.0018); left ventricle end diastolic volume (LVedVol) was decreased (CA 78.86 ± 33.27 vs. nCA 111.20 ± 27.89 mL; *p* < 0.0001). As a consequence, left atrial volume (LAV) was increased in CA in both absolute values (CA 95.04 ± 43.45 vs. nCA 59.00 ± 21.84 mL; *p* = 0.0108) and when indexed for body surface area (LAVi; CA 55.48 ± 23.02 vs. nCA 32.68 ± 10.72 mL/m^2^; *p* = 0.0271) and height elevated to 2.7 (LVMih^2,7^; CA 24.60 ± 10.80 vs. nCA 14.15 ± 5.19 gr/m^2,7^; *p* = 0.031) (Table 4).

Heart ultrasound images in CA patients revealed an inhomogeneity of the interventricular septum, that appeared as “ground glass” in 20/54 patients or “sparkling” in 28/54 patients. All Left ventricles appeared as hypertrophic. Normal contractive function was also described in 10/54 CA patients but more frequently, the heart showed global or segment hypokinesia (28/54), segmental akinesia (4/54) or dyskinesia (4/54), whereas 6/54 patients had a combination of different patterns. Three patients also showed dilated myocardiopathy with marked wall hypertrophy.

Mitral Doppler analysis of blood in-flow (Figure 1) revealed an increased E velocity peak (CA 71.69 ± 25.65 vs. nCA 57.40 ± 14.27 cm/s; *p* = 0.0085) while no significant difference was found in the A velocity. Diastolic dysfunction was constant in CA with an increased incidence of a severe pattern (*p* < 0.0001). Similarly, e’ velocity of the Tissue Doppler Imaging (TDI) analysis result dramatically decreased (CA 4.79 ± 2.79 vs. nCA 8.33 ± 2.73 cm/s; *p* = 0.0095) with a clear increase in the E/e’ ratio (CA 29.37 ± 29.13 vs. nCA 7.03 ± 0.57; *p* = 0.081)

Right heart involvement is described as a consequence of IVS amyloid deposition [23,24]. We found a significant increase in the right atrium (RA) in CA patients, measured as biplane area (CA 22.22 ± 6.49 vs. nCA 19.00 ± 3.89 cm^2^; *p* = 0.0179). Similar to this finding, there was an increase in the Right ventricle (RV) basal diameter (RVd1) (CA 37.39 ± 9.39 vs. nCA 33.22 ± 3.06 mm; *p* = 0.047) and the diameter of the inferior cava vein (IVC) at rest (CA 18.39 ± 5.81 vs. nCA 15.33 ± 4.89 mm; *p* = 0.011). We also found an increase in RV wall thickness (RVWt) in CA (CA 9.87 ± 1.73 vs. nCA 7.00 ± 1.05 mm; *p* = 0.0001), resulting in a reduced total RV volume evaluated as biplane area (CA 19.91 ± 7.37 vs. nCA 22.83 ± 5.98 cm^2^; *p* = 0.0317). RV also showed a reduced systolic function. To achieve its quantification, we measured the tricuspid annular plane excursion (TAPSE). This parameter proved to be reduced in CA compared to nCA patients (CA 18.73 ± 8.32 vs. nCA 26.58 ± 1.73 mm; *p* = 0.0317). Finally, the continuous-wave (CW) evaluation of tricuspid regurgitation velocity (TRV) did not reveal any difference between patients affected and not affected by CA (CA 2.24 ± 0.47 vs. nCA 2.22 ± 0.45 mm; *p* = ns) despite the fact that semi-quantitative evaluation of the estimated pulmonary arterial pressure (PAPs) was increased in CA (CA 38.27 ± 10.67 vs. nCA 28.38 ± 3.75 mmHg; *p* = 0.0053) (Table 4).

We examined the data of all 135 patients for a possible correlation between right heart parameters and immunological patterns. TAPSE indicated a significant positive correlation with lymphocyte count (r = 0.47; *p* = 0.031), Gamma globulins (r = 0.43, *p* = 0.033), Monoclonal component (r = 0.72; *p* = 0.047) and IgG values (r = 0.62, *p* = 0.018). At the same time, this parameter showed a significant negative correlation with LDH (r = −0.57, *p* = 0.005), IVS (r = −0.51, *p* = 0.008) and diastolic function evaluated as E/e’ (r = −0.60, *p* = 0.003) (Table 5). RA area, IVC diameter and PAPs were correlated only to E/e’ ratio (Table 5). Only PAPs were correlated to Beta2microblobulin value (r = −0.41, *p* = 0.043) while no other correlation was found between ICV, PAPs, TRV and RA area and the parameters examined (Table 5). On the contrary, RVd1 was positively correlated to the monoclonal component (r = 0.67, *p* = 0.048). Cardiac biomarkers present different correlations. NT-proBNP and TnI were negatively correlated to the monoclonal component (Table 5) but only NT-proBNP also presented a negative correlation to lymphocyte count, while the TnI result directly correlated to LDH. Finally, both NT-proBNP and TnI were directly correlated to IVS values but only NT-proBNP was also directly correlated to E/e’ ratio (r = 0.57, *p* = 0.013).

Cardiac biomarkers proved to be differently related to RH parameters. Troponin I did not correlate with any measure performed on RH. NT-proBNP proved positively related to RV wall thickness (RWt) (r = 0.61, *p* = 0.020) and to indirect evaluation of RV pressure overload such as IVC diameter (r = 0.64, *p* = 0.007) and PAPs (r = 0.43, *p* = 0.036). LDH was showed to be negatively related to RV area (r = −0.47, *p* = 0.049). Both NT-proBNP and LDH were negatively related to TAPSE (respectively, r = −0.52, *p* = 0.028 and r = −0.58, *p* = 0.005), while there was no correlation between cardiac and RA areas, TRV and RVd1 (Table 6).

To assess whether RH findings might be associated to LH involvement we performed a correlation between the main parameters of the two sides of the heart. We found that right heart parameters presented different correlations to LH measurements. TAPSE did not present any significant correlation other than the ones previously described (Table 5 and Table 7). Apart from the correlation with E/e’ ratio (Table 5), no other correlation was found between PAPs and other LH parameters (Table 5 and Table 7), while TRV had no correlation at all. ICV and RA area were positively correlated with LVM, volumes of LA and LV, and IAS thickness as well as with E/e’ ratio, while no correlation was found with EF% (Table 7). RVd1 was positively correlated with IVS (r = 0.39, *p* = 0.049), LVM (r = 0.63, *p* = 0.001), IAS (r = 0.47, *p* = 0.027) and presented a negative correlation with EF% (r = −0.37, *p* = 0.049), while no correlation was found with E/e’. On the contrary, RWt presented no correlation with LV mass, volume and EF%, but was positively correlated with E/e’ (Table 7). Finally, RV area was positively correlated not only with volumes of LA (r = 0.39, *p* = 0.049) and LV (r = 0.57, *p* = 0.006) but also with IAS thickness (r = 0.54, *p* = 0.008) (Table 7).

**Figure 3:**
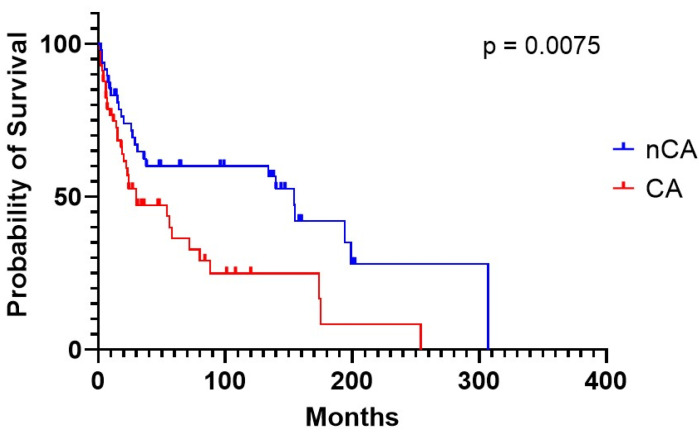
Survival of CA versus non-CA patients.

**Figure 4:**
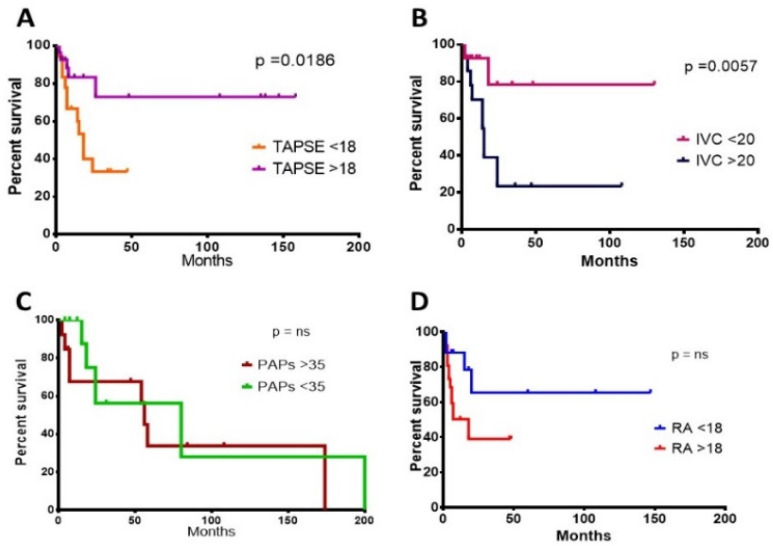
Survival of CA patients according to the different Right Heart parameters cut-off: TAPSE < 18 mm (**A**); IVC diameter < 20 mm (**B**); PAPs < 35 mmHg (**C**) and RA area < 18 cm^2^ (**D**). ns = non significant.

**Figure 5:**
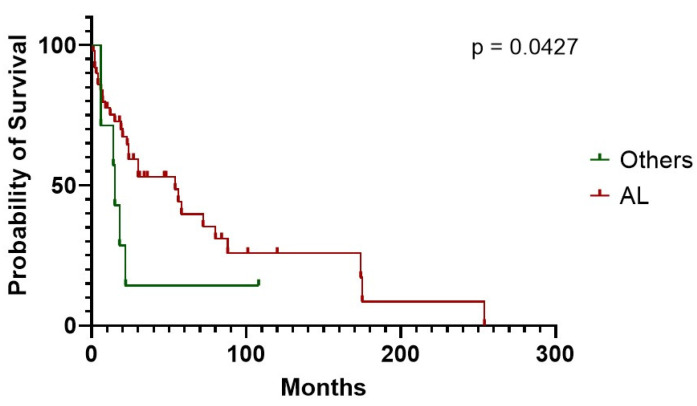
Survival of CA patients affected by AL amyloidosis compared to other amyloid types.

**Figure 6:**
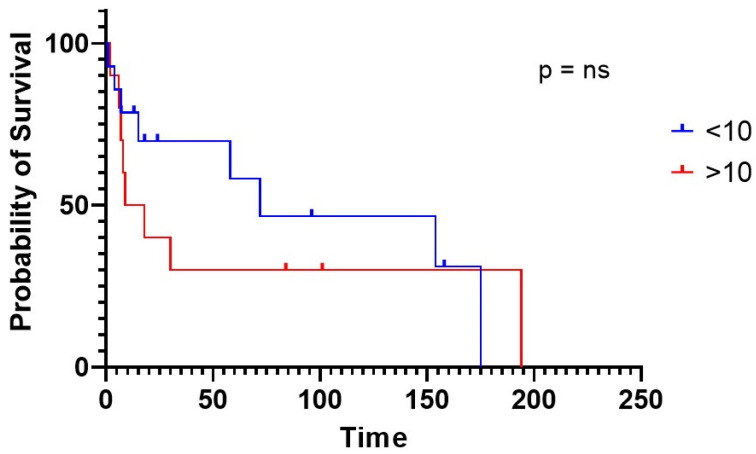
Survival of CA patients affected by AL amyloidosis evaluating bone marrow plasmocytosis % using 10% as value for each boundary class. ns = non significant

CA had very poor survival compared to controls (30 vs. 154 months in CA vs. nCA, respectively, *P* = 0.0.075) (Figure 3). CA individuals experienced a worse mean survival also when stratified according to NT-proBNP levels, using 2500 pg/mL as class boundary (174 vs. 5.5 months, for patients below vs. above the median, respectively, *p* < 0.0001). Similar results were obtained when stratifying CA patients according to TnI levels and using 0.10 ng/mL as class boundary (254 vs. 15 months, for patients below vs. above the median, respectively, *P* = 0.049). In much the same way, decreased TAPSE, an indirect expression of right heart systolic function, was correlated with worse prognosis (18.0 months median survival vs. not reached in subjects above 18 mm, respectively, *p* = 0.0186). Moreover, patients showed a lower life expectancy with an increased inferior cava vein diameter, considering 20 mm maximum diameter as class boundary (15.6 months median survival vs. not reached in subjects above 20 mm, respectively, *P* = 0.0180). The above findings are the only two right heart parameters that proved to be indicative of a worse prognosis (Table 8, Figure 4). CA survival differs according to treatment possibility: AL involvement presents a longer survival compared to other Amyloid types (54 vs. 15 months median in AL subjects vs. other types, *p* = 0.0427) (Figure 5). In AL-CA we found an increased survival using 10% as value for bone marrow plasmocytosis % (BM-PC%) class boundary (72 vs. 13.5 months, for patients above the median, respectively, *P* = ns) but this value was not significantly different (Figure 6).

## 4. Discussion

Cardiac involvement represents a truncal clinical event, impacting amyloidosis clinical outcome [25,26]. Left ventricular damage has been reported as a major pathophysiological determinant of heart dysfunction in CA [27]. Conversely, scanty data describe right heart evaluation when evaluating the patient with amyloidosis [24,28]. Remarkably, the current evidence pinpoints [29,30] the relevance of global heart involvement, with both left and right systolic impaired function. Our data corroborated the available findings and highlighted that the right-heart volume as well as the pressure-measurements seem not to be a mere direct consequence of the left-ventricle dysfunction, but the potential effect of a bona fide de novo amyloid-induced damage. Of note, we did not find a correlation between RH parameters and a specific biomarker of LV damage, namely troponin. There was no correlation with increased pulmonary pressure or TRV and IVS. One of the parameters related to RH alteration remains the diastolic dysfunction. Thus, RV damage appears not to be completely unrelated to the underlying LV involvement. In fact, also considering the diastolic dysfunction, the lack of correlation between TAPSE and LH parameters indicate that RV dysfunction relates to primary damage of RH and a consequence of LV dysfunction. In particular, it is possible to speculate about an increase of LV pre-load as a consequence of LV dysfunction, but had it been a consequence of this mechanism, a RV volume over-load in CA should be found. On the contrary, the lack of increase in RV volume could be a consequence of a venous overload. The increase in RVWt could also induce ventricle stiffness and a reduction in RV volume in RH. Moreover, the increased RV wall thickness did not correlate to LV thickness. Therefore, lack of a direct correlation between RV and LV damage can be proposed. Hence, the final stage is severe global heart failure. Our data point towards a vicious cycle driven by right heart damage on top of the global cardiac dysfunction. Nonetheless, according to our results and consistently with literature evidence, it is tempting to speculate that the different, and dismal clinical impact in non-AL CA might be explained by the right-ventricle involvement [6,31]. Specifically, the progression of disease relates to TAPSE depression. LV pre-load increase proceeds side by side with RH systolic dysfunction. The latter is also associated to RH diastolic dysfunction, inducing a volume and pressure over-load on the venous system. Finally, a reduction of LV pre-load accelerates heart disease and eventually induces low-range heart failure. Our hypothesis-generating findings warrant further validation aiming to dissect novel potential druggable mechanisms that could satisfy the unmet medical need to approach CA involvement in non-AL-diseased patients. We also observed an increase in BUN and proteinuria, quite likely related to progressive kidney damage within our overall patients’ cohort. Given that several staging systems for renal outcome have attempted to predict the dialysis risk over time [32], it is reasonable to highlight that along with the already validated stratification systems based on eGFR and proteinuria levels, BUN evaluation warrants inclusion in the renal damage severity stepwise scoring.

An ancillary finding, from a pathophysiologic standpoint, is the production of highly dynamic light chains that overcome the chaperon’s ability to control the misfolding while a dynamic interaction with the matrix component, shear forces and endoproteinases lead to oligomers. These molecules display toxicity, organ dysfunction and reduced survival formation [33,34,35]. More precisely, the cardiac toxicity seems to be related to p38MAPK reactive oxygen species production, cellular dysfunction, cardiomyocyte death and NT-proBNP transcription [36,37]. Our data confirm increased serum NT-proBNP and BNP as biomarkers of cardiac dysfunction [38,39]. Moreover, NT-proBNP, deemed to be the most sensitive marker of heart involvement in AL amyloidosis, is also used for tumor staging and cardiac response assessment [40]. Of note, our data significantly extend the diagnostic and prognostic value of NT-proBNP, directly correlated to PAPs, RVd1; furthermore, IVC has been deemed clinically valuable when stratifying right heart involvement. Of note, a survival model based on high-sensitive cardiac troponin (hs-cTn) seems to improve the prognostic staging of patients with AL amyloidosis [40] and might suffice to better stratify right heart involvement with severe features. Monitoring the population at risk when detecting MGUS with abnormal kappa/lambda ratio using cardiac (NT-proBNP) and renal (urinary albumin) biomarkers has been shown to be effective in AL amyloidosis [41,42], but scanty evidence supports the value of risk and prognostic stratification for non-AL amyloidosis. In this framework, we considered LDH levels to be a suitable candidate for biochemical profiling of right heart involvement, being associated with both cardiac and liver damage [43]. Since data regarding elderly populations are mainly limited to AL patients’ cohorts [44], novel biological markers can open novel diagnostic and therapeutic windows among non-AL amyloidosis. Dispenzieri et al. [45] used soluble suppression of tumorigenicity 2 (sST2) to prognosticate patients with a worse clinical outcome with AL amyloidosis independently of NT-proBNP and hs-cTn, by mirroring the cardiac remodeling and fibrosis. The results showed impaired heart function, inducing endothelial damage as well. The volume over-load is the main driver of the above-mentioned mechanism. Therefore, ultrasound is a useful tool proposed to assess the volume status in cardiovascular disease [46]. Our data corroborated those findings, pointing towards an increased IVC diameter as well as RA area. However, fibrosis will be induced by amyloid deposition in the ventricle wall, prompting the systolic dysfunction found in the form of decreased TAPSE. Additionally, Kastritis et al. [47] described elevated serum levels of VWF antigen to be relevant in predicting endothelial dysfunctions. In line with those data, our results regarding the platelet levels emphasize the VWF relevance in the aggregation of platelets and their adhesion to subendothelial cells, while enhancing the risk of atherosclerosis and potentially contributing to a worse cardiovascular outcome. In fact, due to the increase in RV pre-load, the increase in shear stress will induce endothelial damage [48,49]. Consequently, an impaired microcirculation [50] can trigger a PLT activation [51]. Moreover, since the growth differentiation factor-15 (GDF-15), a member of the TGF-beta family, has been described to be controlled by MAP kinase and amyloid, along with increased inflammation, oxidative stress, hypoxia and oncogene activation, it is reasonable to foresee the direct role played by GDF-15 in fueling the multiorgan and renal dysfunction in both AL [52] and non-AL amyloidosis.

Finally, from an immunological perspective, immunoparesis in newly diagnosed AL amyloidosis represents a marker for response and survival along with the increased risk of infections; immunoparesis has been shown to be a response and survival marker and patients with full suppression of the uninvolved immunoglobulins are less likely to achieve an optimal response [53]. Moreover, immunoparesis defined by heavy and light chain suppression is a marker of long-term outcomes in cardiac involvement [54]. Hence, our original finding regarding the increased NLR might support the characterization of a particularly high risk immunoparetic phenotype [55] with right heart involvement warranting immune reconstitution as in other secondary hypogammaglobulinemia [56,57].

In previous reports, a low plasma cell burden (median 10%) and proliferative rate characterized the amyloidogenic clone [58] and a dismal prognosis was reported for AL patients when >10% of clonal cells are present within the bone marrow [59]. We describe an increased mortality in CA subjects, confirming the above-mentioned trend; however, we did not find a statistically significant difference. Secondarily, it is well known that the persistence of the clone, assessed by multiparametric flow cytometry in AL amyloidosis, is linked to poor PFS [60], and the reduction of this clone is associated with an improved cardiac and renal response [61].

This study has clear limitations, due to the retrospective design and the sample size. Weaknesses warrant future statistically powered studies in order to evaluate, in a proper prospective analysis, the impact on clinical outcome in terms of survival. Another consequence of the retrospective nature of this study is the outdated classification of Amyloid substance origin. In fact, although the amyloid diagnosis was histologically made, there was no possibility to have a second-look analysis of the samples taken before 1999. Regarding AL amyloidosis, the presence of a small, but dangerous, B cell clone producing misfolded free light chains [62] stimulates a comprehensive biological dissection, able to translate the complex genomic architecture into the real-life clinical disease taxonomy [63], paralleling the biological insights gained in multiple myeloma [64,65]. Finally, due to the retrospective nature of the study, to achieve a homogeneity it was not possible to include some data obtained using more recent ultrasound techniques such as LV strain analysis or three-dimension volume measurements.

Overall, our and previous data prompt deeper basic research aiming to understanding the mechanisms of disease and heart involvement in both AL and non-AL amyloidosis, in order to efficiently identify biomarkers and imaging-based diagnostic tools that may quite likely improve disease management.

## Figures and Tables

**Table 1:** Patients’ general characteristics. Ns = non significant

	CA	nCA	*p*-Value
Sex M (%)	29 (53.70)	41 (50.62%)	Ns
Age (years)	63.95 ± 12.82	58.33 ± 15.65	Ns
Weight (kg)	63.64 ± 12.01	67.08 ± 9.90	Ns
Height (cm)	165.00 ± 7.48	169.3 ± 9.18	Ns
BMI (kg/m^2^)	19.25 ± 3.41	19.82 ± 2.82	Ns
BSA (m^2^)	1.70 ± 0.17	1.77 ± 0.15	Ns
SBP (mmHg)	118.20 ± 13.05	125.00 ± 12.25	0.0326
DBP (mmHg)	72.92 ± 8.31	73.13 ± 5.94	Ns
Heart Rate (bpm)	75.26 ± 14.20	76.44 ± 14.13	Ns
AL amyloidosis	44 (81.48%)	62 (76.54%)	Ns

**Table 2:** Hematological and immunological parameters between the groups of patients. Ns = non significant

	CA	nCA	*p*-Value
**Hemoglobin (g/dL)**	12.08 ± 2.04	12.11 ± 2.30	Ns
**Mean Corpuscular Volume (fL)**	90.18 ± 6.54	90.79 ± 8.31	Ns
**White Blood Cells (×10^3^ cell/dL)**	6.89 ± 2.56	6.79 ± 2.34	Ns
**Neutrophils (×10^3^ cell/dL)**	4.64 ± 2.60	4.02 ± 2.37	Ns
**Neutrophils (%)**	68.68 ± 11.70	65.03 ± 10.33	Ns
**Lymphocytes (×10^3^ cell/dL)**	1.40 ± 0.94	1.55 ± 0.90	Ns
**Lymphocytes (%)**	22.53 ± 10.41	26.39 ± 9.84	Ns
**Lymphocytes CD20+ (%)**	13.18 ± 15.11 (44 pz)	2.00 ± 1.41 (62 pz)	0.0110
**Platelets (×10^3^ cell/dL)**	221.60 ± 113.30	246.40 ± 93.50	0.0038
**Neutrophil to Lymphocyte ratio (NLR)**	4.25 ± 3.63	3.12 ± 2.05	0.0307
**Platelet to Lymphocyte ratio (PLR)**	183.1 ± 118.0	164.4 ± 95.75	Ns
**Erythrocyte Sedimentation Rate (ESR) (mm1 h)**	44.54 ± 13.42	51.82 ± 39.01	Ns
**C-Reactive protein (CRP) (mg/dL)**	12.41 ± 3.17	10.03 ± 3.97	Ns
**Immunoglobulins G (g/dL)**	1.07 ± 8.42	1.49 ± 1.27	0.0493
**Monoclonal Component (g/dL)**	0.99 ± 0.70 (44 pz)	1.37 ± 1.00 (62 pz)	Ns
**Monoclonal Component (%)**	14.47 ± 9.04 (44 pz)	17.51 ± 10.33 (62 pz)	Ns
**Free Light Chain (FLC) λ**	380.0 ± 166.4 (16 pz)	369.4 ± 140.2 (19 pz)	Ns
**Free Light Chain (FLC) k**	109.9 ± 67.70 (164 pz)	65.94 ± 69.74 (19 pz)	Ns
**Beta2microglobuline**	6.74 ± 2.27	4.11 ± 2.35	Ns
**BM Plasmocytosis (%)**	13.61 ± 14.71 (44 pz)	16.31 ± 11.02 (62 pz)	Ns

**Table 3:** Laboratory parameters in cardiac Amyloidosis (CA) and non-CA patients. Ns = non significant

	CA	nCA	*p*-Value
**Albumin (g/dL)**	3.05 ± 0.76	3.15 ± 1.11	Ns
**Total proteins (g/dL)**	6.06 ± 0.17	6.62 ± 1.69	Ns
**Creatinine (mg/dL)**	1.39 ± 1.31	1.21 ± 0.68	Ns
**eGFR (mg/min)**	67.63 ± 31.10	64.30 ± 31.71	Ns
**BUN (mg/dL)**	63.14 ± 43.46	45.30 ± 24.31	0.0314
**Proteinuria (mg/24 h)**	2467.3 ± 574.0	4481.2 ± 876.3	0.0383
**Sodium (mEq/L)**	139.4 ± 3.65	140.20 ± 3.72	Ns
**Potassium (mEq/L)**	4.23 ± 0.67	4.31± 0.54	Ns
**LDH (U/L)**	219.4 ± 94.21	181.8 ± 71.76	0.0011
**CPK (U/L)**	80.31 ± 55.83	76.40 ± 35.22	Ns
**NT-proBNP (pg/mL)**	8633.2 ± 2636.1	1875.5 ± 850.8	0.031
**Troponin I (ng/mL)**	9.13 ± 5.79	0.19 ± 0.37	0.047

**Table 4:** Echocardiographic parameters in CA and non-CA patients. Ns = non significant

	CA	nCA	*p*-Value
**Ejection Fraction (EF) (%)**	52.60 ± 10.58	60.94 ± 4.44	0.0018
**Left atrium Vol. (LAV) (mL)**	95.04 ± 43.45	59.00 ± 21.84	0.0108
**LAVi (mL/m^2^)**	55.48 ± 23.02	32.68 ± 10.72	0.0271
**LAVih^2,7^ (mL/m^2,7^)**	24.60 ± 10.80	14.15 ± 5.19	0.0310
**Interventricular septum (IVS) (mm)**	15.71 ± 3.21	11.03 ± 2.04	<0.0001
**Left ventricle diameter (LVedD) (mm)**	43.80 ± 8.39	50.17 ± 4.32	0.0143
**Posterior wall diameter (PWd) (mm)**	15.39 ± 2.97	10.97 ± 2.06	<0.0001
**Left Ventricle Mass (LVM) (g)**	296.2 ± 112.5	166.0 ± 108.1	0.0151
**LVMi (g/m^2^)**	184.4 ± 64.01	129.9 ± 47.47	0.0132
**LVMih^2,7^ (mL/m^2,7^)**	79.81 ± 27.10	56.75 ± 25.06	0.0191
**Left Ventricle end-diastolic Volume (LVedVol) (mL)**	78.86 ± 33.27	111.2 ± 27.89	<0.0001
**Relative wall thickness (RWT)**	0.72 ± 0.29	0.40 ± 0.15	0.0022
**Inter atrial septum (IAS) (mm)**	8.75 ± 2.09	4.87 ± 0.84	0.0001
**Right atrium area (cm^2^)**	22.22 ± 6.49	19.00 ± 3.89	0.0179
**Inferior cava vein (ICV) (mm)**	18.39 ± 5.81	15.33 ± 4.89	0.0112
**Right ventricle basal diameter (RVd1) (mm)**	37.39 ± 9.39	33.22 ± 3.06	0.0471
**Right ventricle wall thickness (RVWt) (mm)**	9.87 ± 1.73	7.09 ± 1.04	0.0001
**Right ventricle area (cm^2^)**	19.91 ± 7.37	22.83 ± 5.98	0.0317
**Tricuspid Annulus plan excursion (TAPSE) (mm)**	18.73 ± 8.32	26.58 ± 1.73	0.0495
**Tricuspid Regurgitation velocity (TRV) (cm/s)**	3.03 ± 0.47	2.22 ± 0.45	Ns
**Estimated Pulmonary Arterial Pressure (PAPs) (mmHg)**	38.27 ± 10.67	28.38 ± 3.75	0.0053
**Velocity E (cm/s)**	71.69 ± 25.65	57.40 ± 14.27	0.0085
**Velocity A (cm/s)**	53.86 ± 24.72	63.17 ± 25.70	Ns
**E/A**	1.75 ± 1.96	1.02 ± 0.49	0.0435
**Velocity e’ (cm/s)**	4.79 ± 2.79 (31 pz)	8.33 ± 2.73 (55 pz)	0.0095
**E/e’**	29.37 ± 29.13	7.03 ± 0.57	0.0081
**Diastolic Dysfunction (none/I/II/III)**	0/22/8/24	25/51/5/0	<0.0001

**Table 5:** Correlation between right heart parameters and cardiac biomarkers with right heart and immunological parameters. Ns = non significant

	Lymphocyte Count		Gamma Globulins		Monoclonal Component		IgG		LDH		B2M		IVS		E/e’	
	r	*p*	r	*p*	r	*p*	r	*p*	r	*p*	r	*p*	r	*p*	r	*p*
**TAPSE**	0.47	0.031	0.43	0.033	0.72	0.047	0.62	0.018	−0.57	0.005	−0.13	ns	−0.51	0.008	−0.60	0.003
**ICV**	−0.30	Ns	0.16	Ns	−0.19	Ns	−0.33	Ns	0.21	ns	−0.08	ns	0.35	Ns	0.49	0.029
**PAPs**	−0.03	Ns	0.05	Ns	0.07	Ns	0.11	Ns	0.19	ns	−0.41	0.043	0.03	Ns	0.43	0.043
**TRV ^#^**	0.07	Ns	0.06	Ns	0.16	Ns	−0.21	Ns	−0.14	ns	−0.33	ns	0.17	Ns	−0.05	ns
**RA area**	−0.03	Ns	0.23	Ns	−0.39	Ns	−0.14	Ns	−0.03	ns	0.29	ns	0.31	Ns	0.45	0.045
**RVd1**	−0.31	Ns	0.14	Ns	0.67	0.048	−0.19	Ns	0.01	ns	−0.08	ns	0.40	0.049	−0.20	ns
**RWt ^#^**	−0.24	Ns	−0.03	Ns	−0.03	ns	−0.20	Ns	0.26	ns	−0.33	ns	0.34	Ns	0.69	0.007
**NT-proBNP ^#^**	−0.46	0.015	0.04	Ns	−0.47	0.047	−0.38	Ns	0.02	ns	0.17	ns	0.43	0.022	0.57	0.013
**TnI ^#^**	−0.15	Ns	−0.33	Ns	−0.57	0.049	−0.33	Ns	0.60	0.001	0.04	ns	0.44	0.023	−0.13	ns

^#^ indicate results analyzed using non-parametric Spearman correlation analysis. Remaining were correlated using Pearson test.

**Table 6:** Correlation between right heart parameters with cardiac biomarkers. Results were obtained with Spearman correlation Analysis. Ns = non significant

	NT-proBNP		LDH		TnI	TnI
	R	*p*	r	*p*	R	*p*
**TAPSE**	−0.52	0.028	−0.58	0.005	0.30	Ns
**ICV**	0.64	0.007	0.07	Ns	0.32	Ns
**PAPs**	0.43	0.036	0.29	Ns	0.15	Ns
**TRV**	0.07	Ns	0.05	Ns	−0.03	Ns
**RA area**	0.19	Ns	−0.18	Ns	0.16	Ns
**RVd1**	0.32	Ns	−0.29	Ns	0.32	Ns
**RV Area**	0.20	Ns	−0.47	0.049	0.16	Ns
**RWt**	0.61	0.020	0.32	Ns	−0.06	Ns

**Table 7:** Correlation between Right and Left cardiac parameters. Ns = non significant

	IVS		LVM		LA Volume		LVedVol		IAS		EF%		E/e’	
	r	*p*	r	*p*	r	*p*	r	*p*	r	*p*	R	*p*	r	*p*
**TAPSE**	−0.51	0.008	−0.24	Ns	−0.07	ns	0.18	ns	−0.24	ns	0.35	Ns	−0.60	0.003
**ICV**	0.35	ns	0.54	0.008	0.54	0.008	0.18	ns	0.54	0.008	−0.31	Ns	0.49	0.029
**PAPs**	0.03	ns	0.04	ns	0.31	ns	0.20	ns	0.17	ns	−0.14	Ns	0.43	0.043
**TRV**	0.16	ns	0.10	ns	0.13	ns	0.02	ns	0.06	ns	−0.12	Ns	−0.05	ns
**RA area**	0.32	ns	0.68	0.0003	0.60	0.002	0.50	0.015	0.67	0.014	0.01	Ns	0.45	0.045
**RVd1**	0.39	0.049	0.63	0.001	0.33	ns	0.23	ns	0.47	0.027	−0.37	0.049	−0.20	ns
**RV Area**	0.23	ns	0.59	ns	0.39	0.049	0.57	0.006	0.54	0.008	−0.11	Ns	−0.06	ns
**RWt**	0.23	ns	0.16	ns	0.34	ns	−0.32	ns	0.25	ns	0.10	Ns	0.69	0.007

**Table 8:** Predictor of mortality in Cardiac Amyloidosis (Mantel–Haenszel). Ns = non significant

	HR	*p*-Value
**TAPSE < 18 mm**	3.37	0.0186
**ICV > 20 mm**	4.86	0.0057
**PAPs > 35 mmHg**	1.53	Ns
**RA area > 18 cm^2^**	1.84	Ns
**RVd1 >7 mm**	1.37	Ns
**E/e’ > 10**	9.63	0.038
**NT-proBNP > 2500 ng/mL**	18.47	0.0001
**TnI > 0.1 pg/mL**	5.81	0.049
